# Supplementary material for: The Systems Biology Research Tool: evolvable open-source software
Source: BMC Syst Biol. 2008 Jun 29;2:55. doi: 10.1186/1752-0509-2-55 (PMC2446383; doi:10.1186/1752-0509-2-55)
Supplement: Additional file 1 — SBRT Archive. An archive of the current version of the Systems Biology Research Tool. [file 1752-0509-2-55-S1.zip › sbrt-1.4.0/doc/developers_guide/api/sbrt/kernel/math/stat/comp/KendallTau.html]

KendallTau


|  |  |  |
| --- | --- | --- |
| **Overview** \| **Package** \| **Class** \| **Use** \| **Tree** \| **Deprecated** \| **Index** \| **Help** | |  |
| **PREV CLASS**   **NEXT CLASS** | **FRAMES**    **NO FRAMES**     **All Classes** |  |
| SUMMARY: NESTED \| FIELD \| CONSTR \| METHOD | DETAIL: FIELD \| CONSTR \| METHOD |  |


---


## sbrt.kernel.math.stat.comp Class KendallTau<V>

```
java.lang.Object
  sbrt.kernel.math.stat.comp.KendallTau<V>
```

**Type Parameters:**: `V` - the variable type.

**All Implemented Interfaces:**: Correlator<V,TauResult>

**Direct Known Subclasses:**: KendallTauA, KendallTauB

---

``` public abstract class KendallTau<V> extends java.lang.Object implements Correlator<V,TauResult> ```

This abstract class is a skeleton implementation of a Kendall's tau
correlation statistic computer. It is implemented using algorithms from the
book *Rank Correlation Methods, 5th Edition* by Kendall & Gibbons.

**Author:**
:   This class was written and documented by Jeremiah Wright while in the
    Wagner lab.

---

**Constructor Summary**

`KendallTau()`


| **Method Summary** | |
| --- | --- |
| `TauResult` | `corr(java.util.Collection<java.lang.Double> xValues, java.util.Collection<java.lang.Double> yValues)`             Computes and returns the correlation results for the provided values. |
| `TauResult` | `corr(double[] xValues, double[] yValues)`             Computes and returns the correlation results for the provided values. |
| `TauResult` | `corr(java.lang.Double[] xValues, java.lang.Double[] yValues)`             Computes and returns the correlation results for the provided values. |
| `TauResult` | `corr(java.util.Map<? extends V,java.lang.Double> xValues, java.util.Map<? extends V,java.lang.Double> yValues)`             Computes and returns the correlation results for the provided values. |
| `double` | `getApproxZ(java.util.Collection<java.lang.Double> xValues, java.util.Collection<java.lang.Double> yValues)`             Computes and returns a Z approximation for the provided values. |
| `double` | `getApproxZ(double[] xValues, double[] yValues)`             Computes and returns a Z approximation for the provided values. |
| `double` | `getApproxZ(java.util.Map<? extends V,java.lang.Double> xValues, java.util.Map<? extends V,java.lang.Double> yValues)`             Computes and returns a Z approximation for the provided values. |
| `int` | `getS(java.util.Collection<java.lang.Double> xValues, java.util.Collection<java.lang.Double> yValues)`             Computes and returns the S value of the provided values. |
| `int` | `getS(double[] xValues, double[] yValues)`             Computes and returns the S value of the provided values. |
| `int` | `getS(java.util.Map<? extends V,java.lang.Double> xValues, java.util.Map<? extends V,java.lang.Double> yValues)`             Computes and returns the S value of the provided values. |
| `double` | `getTau(java.util.Collection<java.lang.Double> xValues, java.util.Collection<java.lang.Double> yValues)`             Computes and returns the correlation statistic for the provided values. |
| `abstract  double` | `getTau(double[] xValues, double[] yValues)`             Computes and returns the correlation statistic for the provided values. |
| `double` | `getTau(java.util.Map<? extends V,java.lang.Double> xValues, java.util.Map<? extends V,java.lang.Double> yValues)`             Computes and returns the correlation statistic for the provided values. |
| `double` | `getTieScore(java.util.Collection<java.lang.Double> values)`             Computes and returns the tie score of the provided values. |
| `double` | `getTieScore(double[] values)`             Computes and returns the tie score of the provided values. |
| `double` | `getTieScore(java.util.Map<? extends V,java.lang.Double> values)`             Computes and returns the tie score of the provided values. |
| `double` | `getVarS(java.util.Collection<java.lang.Double> xValues, java.util.Collection<java.lang.Double> yValues)`             Computes and returns the variance of S for the provided values. |
| `double` | `getVarS(double[] xValues, double[] yValues)`             Computes and returns the variance of S for the provided values. |
| `double` | `getVarS(java.util.Map<? extends V,java.lang.Double> xValues, java.util.Map<? extends V,java.lang.Double> yValues)`             Computes and returns the variance of S for the provided values. |
| `static void` | `main(java.lang.String[] args)`             Tests the Kendall Tau A and B computers. |

**Methods inherited from class java.lang.Object**

`clone, equals, finalize, getClass, hashCode, notify, notifyAll, toString, wait, wait, wait`

**Constructor Detail**

### KendallTau

```
public KendallTau()
```


**Method Detail**

### getTau

```
public abstract double getTau(double[] xValues,
                              double[] yValues)
```

:   Computes and returns the correlation statistic for the provided values.

    :   **Parameters:**: `xValues` - an ordered array of values.: `yValues` - an ordered array of values. **Returns:**: the correlation statistic for the provided values.

---


### getTau

```
public double getTau(java.util.Map<? extends V,java.lang.Double> xValues,
                     java.util.Map<? extends V,java.lang.Double> yValues)
```

:   Computes and returns the correlation statistic for the provided values.
    The key sets of the provided maps need not be identical, but only the
    intersecting values will be considered.

    :   **Parameters:**: `xValues` - variables mapped to their value.: `yValues` - variables mapped to their value. **Returns:**: the correlation statistic for the provided values.

---


### getTau

```
public double getTau(java.util.Collection<java.lang.Double> xValues,
                     java.util.Collection<java.lang.Double> yValues)
```

:   Computes and returns the correlation statistic for the provided values.

    :   **Parameters:**: `xValues` - an ordered collection of values.: `yValues` - an ordered collection of values. **Returns:**: the correlation statistic for the provided values.

---


### corr

```
public TauResult corr(java.util.Map<? extends V,java.lang.Double> xValues,
                      java.util.Map<? extends V,java.lang.Double> yValues)
```

:   Computes and returns the correlation results for the provided values. The
    key sets of the provided maps need not be identical, but only the
    intersecting values will be considered.

    :   **Specified by:**: `corr` in interface `Correlator<V,TauResult>`
    :   **Parameters:**: `xValues` - variables mapped to their value.: `yValues` - variables mapped to their value. **Returns:**: the correlation results for the provided values.

---


### corr

```
public TauResult corr(java.util.Collection<java.lang.Double> xValues,
                      java.util.Collection<java.lang.Double> yValues)
```

:   Computes and returns the correlation results for the provided values.

    :   **Specified by:**: `corr` in interface `Correlator<V,TauResult>`
    :   **Parameters:**: `xValues` - an ordered collection of values.: `yValues` - an ordered collection of values. **Returns:**: the correlation results for the provided values.

---


### corr

```
public TauResult corr(java.lang.Double[] xValues,
                      java.lang.Double[] yValues)
```

:   Computes and returns the correlation results for the provided values.

    :   **Specified by:**: `corr` in interface `Correlator<V,TauResult>`
    :   **Parameters:**: `xValues` - an ordered array of values.: `yValues` - an ordered array of values. **Returns:**: the correlation results for the provided values.

---


### corr

```
public TauResult corr(double[] xValues,
                      double[] yValues)
```

:   Computes and returns the correlation results for the provided values.

    :   **Parameters:**: `xValues` - an ordered array of values.: `yValues` - an ordered array of values. **Returns:**: the correlation results for the provided values.

---


### getS

```
public int getS(java.util.Map<? extends V,java.lang.Double> xValues,
                java.util.Map<? extends V,java.lang.Double> yValues)
```

:   Computes and returns the S value of the provided values. The key sets of
    the provided maps need not be identical, but only the intersecting values
    will be considered.

    :   **Parameters:**: `xValues` - variables mapped to their value.: `yValues` - variables mapped to their value. **Returns:**: the S value of the provided values.

---


### getS

```
public int getS(java.util.Collection<java.lang.Double> xValues,
                java.util.Collection<java.lang.Double> yValues)
```

:   Computes and returns the S value of the provided values.

    :   **Parameters:**: `xValues` - an ordered collection of values.: `yValues` - an ordered collection of values. **Returns:**: the S value of the provided values.

---


### getS

```
public int getS(double[] xValues,
                double[] yValues)
```

:   Computes and returns the S value of the provided values.

    :   **Parameters:**: `xValues` - an ordered array of values.: `yValues` - an ordered array of values. **Returns:**: the S value of the provided values.

---


### getTieScore

```
public double getTieScore(java.util.Map<? extends V,java.lang.Double> values)
```

:   Computes and returns the tie score of the provided values.

    :   **Parameters:**: `values` - variables mapped to their value. **Returns:**: the tie score of the provided values.

---


### getTieScore

```
public double getTieScore(java.util.Collection<java.lang.Double> values)
```

:   Computes and returns the tie score of the provided values.

    :   **Parameters:**: `values` - a collection of values. **Returns:**: the tie score of the provided values.

---


### getTieScore

```
public double getTieScore(double[] values)
```

:   Computes and returns the tie score of the provided values.

    :   **Parameters:**: `values` - an array of values. **Returns:**: the tie score of the provided values.

---


### getApproxZ

```
public double getApproxZ(java.util.Map<? extends V,java.lang.Double> xValues,
                         java.util.Map<? extends V,java.lang.Double> yValues)
```

:   Computes and returns a Z approximation for the provided values. The key
    sets of the provided maps need not be identical, but only the
    intersecting values will be considered.

    :   **Parameters:**: `xValues` - variables mapped to their value.: `yValues` - variables mapped to their value. **Returns:**: a Z approximation for the provided values.

---


### getApproxZ

```
public double getApproxZ(java.util.Collection<java.lang.Double> xValues,
                         java.util.Collection<java.lang.Double> yValues)
```

:   Computes and returns a Z approximation for the provided values.

    :   **Parameters:**: `xValues` - an ordered collection of values.: `yValues` - an ordered collection of values. **Returns:**: a Z approximation for the provided values.

---


### getApproxZ

```
public double getApproxZ(double[] xValues,
                         double[] yValues)
```

:   Computes and returns a Z approximation for the provided values.

    :   **Parameters:**: `xValues` - an ordered array of values.: `yValues` - an ordered array of values. **Returns:**: a Z approximation for the provided values.

---


### getVarS

```
public double getVarS(java.util.Map<? extends V,java.lang.Double> xValues,
                      java.util.Map<? extends V,java.lang.Double> yValues)
```

:   Computes and returns the variance of S for the provided values. The key
    sets of the provided maps need not be identical, but only the
    intersecting values will be considered.

    :   **Parameters:**: `xValues` - variables mapped to their value.: `yValues` - variables mapped to their value. **Returns:**: the variance of S for the provided values.

---


### getVarS

```
public double getVarS(java.util.Collection<java.lang.Double> xValues,
                      java.util.Collection<java.lang.Double> yValues)
```

:   Computes and returns the variance of S for the provided values.

    :   **Parameters:**: `xValues` - an ordered collection of values.: `yValues` - an ordered collection of values. **Returns:**: the variance of S for the provided values.

---


### getVarS

```
public double getVarS(double[] xValues,
                      double[] yValues)
```

:   Computes and returns the variance of S for the provided values.

    :   **Parameters:**: `xValues` - an ordered array of values.: `yValues` - an ordered array of values. **Returns:**: the variance of S for the provided values.

---


### main

```
public static void main(java.lang.String[] args)
```

:   Tests the Kendall Tau A and B computers.

    :   **Parameters:**: `args` - not required.


---


|  |  |  |
| --- | --- | --- |
| **Overview** \| **Package** \| **Class** \| **Use** \| **Tree** \| **Deprecated** \| **Index** \| **Help** | |  |
| **PREV CLASS**   **NEXT CLASS** | **FRAMES**    **NO FRAMES**     **All Classes** |  |
| SUMMARY: NESTED \| FIELD \| CONSTR \| METHOD | DETAIL: FIELD \| CONSTR \| METHOD |  |


---
